# Temperature-Dependent Plastic Behavior of ASA: Johnson–Cook Plasticity Model Calibration and FEM Validation

**DOI:** 10.3390/ma19030470

**Published:** 2026-01-24

**Authors:** Peter Palička, Róbert Huňady, Martin Hagara

**Affiliations:** 1 Department of Applied Mechanics and Mechanical Engineering, Faculty of Mechanical Engineering, Technical University of Košice, 042 00 Košice, Slovakia; robert.hunady@tuke.sk (R.H.); martin.hagara@tuke.sk (M.H.); 2 Department of Robotics, Faculty of Mechanical Engineering, Technical University of Ostrava, 708 00 Ostrava, Czech Republic

**Keywords:** material model, uniaxial tensile test, numerical model, ASA, FEA, plasticity, Johnson–Cook hardening model

## Abstract

Acrylonitrile Styrene Acrylate (ASA) is widely used in outdoor structural applications due to its favorable mechanical stability and weather resistance; however, its temperature-dependent plastic behavior remains insufficiently characterized for accurate numerical simulation. This study presents a non-standard method of calibrating the temperature-dependent Johnson–Cook (J-C) plasticity model for ASA in the practical operating temperature range below the glass transition temperature. Uniaxial tensile tests at constant strain rate 0.01 s^−1^ were performed at −10 °C, +23 °C, and +65 °C to characterize the effect of temperature on the material’s plastic response. The J-C parameters A, B, and n were identified for each temperature separately and globally using least-squares optimization implemented in MATLAB R2024b, showing good agreement with the experimental stress–strain curves. The calibrated parameters were subsequently implemented in Abaqus 2024 and validated through finite element simulations of the tensile tests. Numerical predictions demonstrated a very high correlation with the experimental data across all temperatures, confirming that the J-C model accurately captures the hardening behavior of ASA. The presented parameter set and calibration methodology provide a reliable basis for future simulation-driven design, forming analysis, and structural assessment of ASA components subjected to variable thermal conditions.

## 1. Introduction

In general, the fundamental mechanical properties of materials—such as the modulus of elasticity, Poisson’s ratio, yield strength, or ultimate strength—which are commonly listed in a material datasheet, are obtained based on standardized tests performed under quasi-static loading conditions at a temperature of 20 °C. In the case of dynamic processes, where the material response depends significantly on the loading rate or on temperature, a more complex description of its behavior is required. Plastics belong to this category of materials. In practice, considerable attention is devoted to determining the mechanical properties of plastics, as evidenced by the large number of specialized publications and scientific articles. A detailed overview of this issue is provided by the authors in their review paper [1], which focuses on the methods used in the dynamic testing of plastics and examines the mechanical properties of several classes of polymers, not only as a function of strain rate and temperature, but also of pressure. Study [2], in turn, provides a comprehensive overview of the methods and devices used in high-speed testing, ranging from drop-weight towers to the split Hopkinson pressure bar and ballistic testing equipment. The work also offers a basic description of the optical methods suitable for the dynamic measurement of displacements and strains, primarily methods based on interferometry, photogrammetry, or thermography. Currently, deformation parameters are predominantly measured using digital video extensometers and systems based on the digital image correlation method.

Plastics are undoubtedly a revolutionary material, and their usability continues to expand—not only in technical practice, but also in the food industry, healthcare, and many other sectors. At present, the production of thermoplastics represents approximately 80% of total plastic production. This is mainly due to their simple preparation and wide applicability in technical practice. In thermoplastics, the molecular chains are held together by relatively weak van der Waals forces. When a thermoplastic is heated, the intermolecular forces weaken, resulting in significant softening of the material. At high temperatures, viscous flow occurs. When the material is cooled, it becomes solid again, retaining its original properties. This cycle of softening with heat and hardening with cooling can be repeated more or less indefinitely. This principle forms the basis of most processing methods for these materials. Its major advantage, but also disadvantage, lies in its temperature dependence, meaning that its mechanical properties change with temperature [3].

Acrylonitrile styrene acrylate (ASA) is an amorphous thermoplastic. Like all styrene-based polymers, ASA is processed mainly by injection-molding, but also by extrusion (sheets and films for thermoforming). Acrylonitrile provides higher strength, toughness, and rigidity compared to standard styrene-based plastics (PS, SAN). It is non-flammable and exhibits moderate thermal and chemical resistance. ASA is probably the most widespread engineering (technical) thermoplastic, giving it a wide range of applications. It is resistant to UV radiation and maintains long-term color stability [4,5,6,7,8].

Thermoplastic polymers exhibit complex deformation mechanisms that fundamentally differ from those of metals, including a strong dependence on strain rate and temperature [9,10,11,12,13,14,15]. Therefore, inverse calibration strategies combining experimental measurements and numerical modeling have become increasingly important for polymer characterization. The accuracy of numerical simulations depends, among other things, on the quality of the input material data that define deformation behavior. Accurate prediction of the mechanical response of ASA components requires reliable constitutive models that capture plastic deformation under various loading conditions. Among the most widely used constitutional models in technical practice are the J-C plasticity model and the Voce model [16,17,18]. In numerical simulations, the predictive capability of finite element analyses is strongly dependent on the proper calibration of material parameters, especially for polymers where strain-rate sensitivity, nonlinearity, and progressive hardening play a dominant role. There is great potential for research on creating constitutive models for various types of materials, including thermoplastics, in various commercial software packages such as VPS, Abaqus, LS-DYNA, and NX [19,20,21,22,23,24]. ASA is commonly applied in the automotive industry, as well as electrical enclosures, sports gear, and building products, because it combines mechanical strength, visual appeal, and strong resistance to color-fading caused by long-term UV exposure. Typical applications include exterior car parts, outdoor furniture, roofing elements, and protective casings for electronic devices used in outdoor environments [25]. For example, in the development of passenger cars, numerical simulations of various static and dynamic tests, including those dependent on temperature, are widely used. The creation of constitutive material models of various polymers is therefore particularly important in this field.

Previous studies [26,27,28] of ASA polymers have predominantly combined extensive experimental characterization with finite element simulations aimed at validating measured mechanical responses and assessing the influence of printing parameters, infill configuration, and microstructural features. In these works, numerical modeling is mainly based on effective or linear elastic (often orthotropic) material descriptions derived from experiments, enabling the accurate reproduction of bending and compression tests as well as global structural behavior. While such approaches provide valuable insight into the role of process-induced anisotropy and geometry, the mechanical response is not described through dedicated constitutive formulations capable of capturing nonlinear, strain-rate-dependent, or thermomechanical effects. Consequently, the development and application of advanced constitutive models specifically for ASA, which would enable predictive simulations under a broader range of loading and environmental conditions, remains an open research topic.

However, the constitutional modeling process can be demonstrated by studies devoted to the creation of material models of ABS polymer. This is a material with similar mechanical properties but lower resistance to weathering and UV radiation. Reference [29] experimentally investigates the mechanical behavior of FDM-printed ABS specimens as a function of layer height, number of layers, raster orientation, and strain rate, showing improved performance with a shorter layer height, fewer layers, and filament alignment along the loading direction. Based on these findings, a novel, thermodynamically consistent continuum constitutive model is developed and calibrated for ABS, accurately predicting strain-rate-dependent and anisotropic behavior under finite deformations, though it is currently only validated for uniaxial loading. In Reference [30] a temperature-dependent model of ABS, divided by the glass transition temperature, was developed using the fitting method to the experimental data. Reference [31] evaluates the ability of four constitutive models to predict the strain-rate- and temperature-dependent deformation of PC/ABS over a wide range of strain rates and temperatures. The modified Mulliken–Boyce model improves accuracy at high strain rates by incorporating evolving internal shear strength, while the Wang adiabatic model provides the most accurate predictions across low, moderate, and high strain rates and temperatures.

In this work, the plastic behavior of ASA is modeled using the Johnson–Cook plasticity model, which captures temperature sensitivity. The objective of this study is to establish and validate a simple yet effective calibration methodology for ASA in the temperature range below the glass transition temperature, which is compatible with standard mechanical testing and suitable for implementation in engineering-scale simulations. The calibrated model is evaluated against independent experimental data to assess its predictive capability. The proposed approach offers an efficient tool for improving the accuracy of mechanical simulations involving ASA and can be extended to other amorphous thermoplastics.

## 2. Materials and Methods

**Figure 1:**
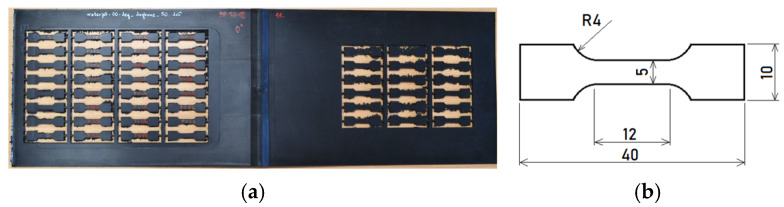
(**a**) Samples cut from ASA plate; (**b**) dimensions of tested samples.

Injection-molding technology was used to produce plates from ASA material with a constant thickness of 2.5 mm. The supplier of material was BASF (Ludwigshafen, Germany) and the material brand used in this study was Luran^®^ S 778 T [32]. For testing purposes, samples (Figure 1b) were cut from plates using a water jet along a specified vector trajectory (Figure 1a).

Uniaxial tensile tests were performed at three temperatures: −10 °C, +23 °C, and +65 °C. This temperature range was based on the needs of the automotive industry, where vehicles or their parts are commonly tested in a temperature range corresponding to typical seasonal changes in the environment between summer and winter. Individual components are thus exposed not only to mechanical load but also to thermal loads. In the case of ASA polymer, these temperatures are below the glass transition temperature.

**Figure 2:**
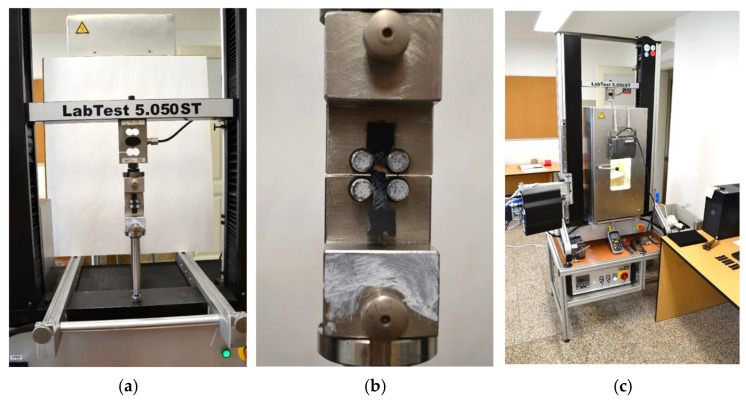
Experimental tests: (**a**) tensile set-up; (**b**) sample after break; (**c**) temperature-controlled chamber.

A universal testing machine LabTest 5.050ST (LABORTECH, Opava, Czech Republic) (Figure 2a) equipped with a temperature-controlled chamber (Figure 2c) was used. The tests were conducted at a constant strain rate of 0.01 s^−1^, consistent with quasi-static loading conditions up to failure (Figure 2b). Strain was measured using a digital video-extensometer focused on the gauge region of the specimen. For each temperature level, seven samples were tested to ensure statistical representativeness and sufficient confidence in the measured mechanical properties with respect to inherent material variability. During testing, samples were conditioned in a temperature chamber to ensure thermal and environmental equilibrium. Liquid nitrogen was used to achieve an ambient temperature of −10 °C.

**Figure 3:**
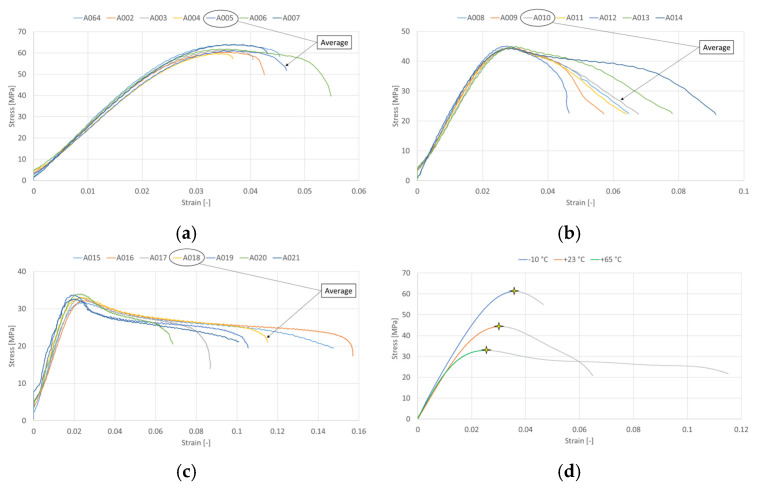
Results from tensile test for (**a**) −10 °C; (**b**) +23 °C; (**c**) +65 °C; and (**d**) average curves for tested temperatures.

Based on the material theory of polymers, it is clear and expected that ASA will exhibit the highest tensile modulus of elasticity (Young’s modulus) at a temperature of –10 °C and the lowest tensile modulus at the highest tested temperature of +65 °C. This was determined from stress–strain curves using linear regression. Poisson’s ratio, which describes the relative transverse contraction to its longitudinal extension, will logically also change with temperature. Since polymers become more ductile as temperature increases, Poisson’s ratio will increase with rising temperature. Poisson’s ratio was not measured experimentally, and for the purpose of the simulations, a constant value of 0.35 was used throughout the tested range of temperatures. According to the supplier, the density of the material ASA was 1.07 g/cm^3^ [32]. Evaluated engineering stress–strain curves are shown in Figure 3.

The obtained engineering stress–strain curves were subsequently converted to true stress–true strain data up to the onset of necking using relations:

(1) $$\varepsilon_{t}=\ln(1+\varepsilon_{e}),\;\sigma_{t}=\sigma_{e}(1+\varepsilon_{e}),$$

where $\varepsilon_{t}$ is true strain, $\varepsilon_{e}$ is engineering strain, $\sigma_{t}$ is true stress, and $\sigma_{e}$ is engineering stress. The points where the curves were trimmed are marked with a star in Figure 3d. Table 1 shows the resulting values of Young’s modulus of elasticity in tension and the standard deviation from the average curves. Since the *R*^2^ value exceeded 0.99 for each curve, we can consider this to be a very good fit.

To achieve a high level of agreement between the experimental and numerical results (see Section 5), it was necessary to appropriately adjust the values of Young’s modulus, which is one of the constants in the material model. Although Young’s modulus does not directly affect the shape of the plasticity curve defined by the J-C model, it does affect the decomposition of the total strain into elastic and plastic components, and thus also affects the position of the plastic part of the curve in the resulting stress–strain diagram. The final values of Young’s modulus used in the FEM calculations are shown in Table 2.

## 3. Constitutive Modeling

To simulate the mechanical behavior of ASA components in finite element analysis (FEA), an accurate plasticity model is required. The J-C model is the industry standard for this purpose, as it captures strain-hardening, temperature-dependent softening, and (for dynamic loading) strain rate effects in a compact, physically meaningful form. However, calibrating the J-C model for thermoplastics like ASA requires careful handling of experimental data and enforcement of physical constraints—unlike metals, thermoplastics exhibit strong, monotonic decreases in stiffness and strength with temperature, and post-necking strain softening that the basic J-C model cannot describe. The model is expressed as follows [33]:

(2) $$\sigma =(A+B\varepsilon^{n})\left(1+C\ln\frac{\dot{\varepsilon }}{{\dot{\varepsilon }}_{0}}\right)\left[1-{\left(T*\right)}^{m}\right]$$

where A is the initial yield stress, B is the strain-hardening modulus, n is the strain-hardening exponent, m is the thermal softening exponent, T* is nondimensional temperature, and ε is the plastic strain. For each individual temperature, the above-mentioned parameters, A, B and n, were optimized. In this case, parameters C and m were not included in the calculation in Abaqus, which simplifies Equation (2) as follows:

(3) $$\sigma =A+B\varepsilon^{n}.$$

**Figure 4:**
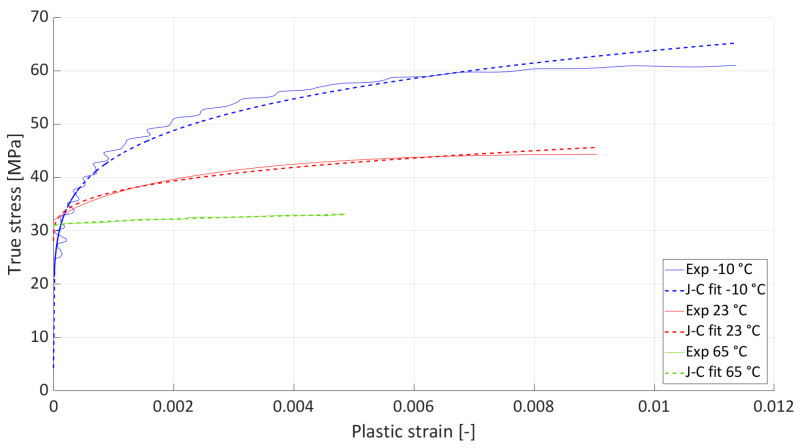
Fit of individual J-C parameters for each temperature.

The plastic strain was computed by subtracting the elastic portion based on the measured elastic modulus. Only the hardening portion up to the ultimate strength was used for constitutive identification. Parameter identification was performed using MATLAB’s nonlinear least-squares solver (lsqcurvefit). The above-mentioned parameters were optimized by minimizing the squared residuals between the model prediction and experimental data. Lower and upper bounds were applied to ensure physical feasibility and numerical stability. Convergence was monitored through changes in both parameters and the cost function, and the goodness of fit was quantified using *R*^2^ and residual analysis. The fit of the J-C parameters to the experimental data is shown in Figure 4, and Table 3 shows the resulting J-C parameter values, including *R*^2^, which is sufficient given that there is significant dispersion in the plastic strain in polymers (see Figure 3).

Parameter A, at a temperature of 263 K, is significantly lower than at other temperatures. This is because, at this low temperature, brittle rather than ductile failure occurs, and there is no significant yield point, at least not in the traditional sense of the word. According to plastics theory, as the temperature decreases, stiffness increases, ductility decreases, and materials become brittle. Optimization showed this to be the most optimal parameter choice.

### Global J-C Model

Instead of calibrating parameters for each temperature individually, MATLAB’s function (lsqnonlin) was used to fit all parameters simultaneously across all datasets. This ensures consistent thermal softening behavior across temperatures. The objective function minimizes weighted residuals to prioritize accurate the fit of the elastic and early plastic regions. At this point, it is necessary to mention how Abaqus expresses parameter T* (nondimensional temperature) in Equation (2):

(4) $$T*\equiv \left\{\begin{array}{c} 0\\ \left(T-T_{\mathrm{transition}})/(T_{\mathrm{melt}}-T_{\mathrm{transition}}\right)\\ 1 \end{array}\right.\;\begin{array}{c} \mathrm{for}\\ \mathrm{for}\\ \mathrm{for} \end{array}\;\begin{array}{c} T<T_{\mathrm{transition}}\\ T_{\mathrm{transition}}\leq T\leq T_{\mathrm{melt}}\\ T>T_{\mathrm{melt}} \end{array},$$

where T is the current temperature, $T_{\mathrm{melt}}$ is the melting temperature, and $T_{\mathrm{transition}}$ is the transition temperature, defined as the one at or below which there is no temperature dependence of the yield stress. Based on Reference [34] the glass transition temperature of ASA is approximately 100 °C (373 K). The tested temperature range was 263 K–338 K, which means that if a value of 373 K were entered into the parameter $T_{\mathrm{transition}}$, all current temperatures would be lower than the transition temperature, meaning that Abaqus would set a zero value for the nondimensional temperature to J-C model. In order to take into account the temperature behavior in the plastic deformation response, it was necessary to adjust Equation (4) so that the glass transition temperature was set to the lowest tested temperature. Since the physical meaning of the parameter $T_{\mathrm{transition}}$ is changing, it is appropriate to introduce a new designation $T_{0}$ instead. This ensures that parameter T* satisfies condition $T_{0}\leq T\leq T_{\mathrm{melt}}$. Parameter $T_{\mathrm{melt}}$ is 240–280 °C based on Reference [32] and for the calculation of global J-C parameters, the middle value of 260 °C (533 K) was chosen. The resulting J-C parameters for the global model are summarized in Table 4.

**Figure 5:**
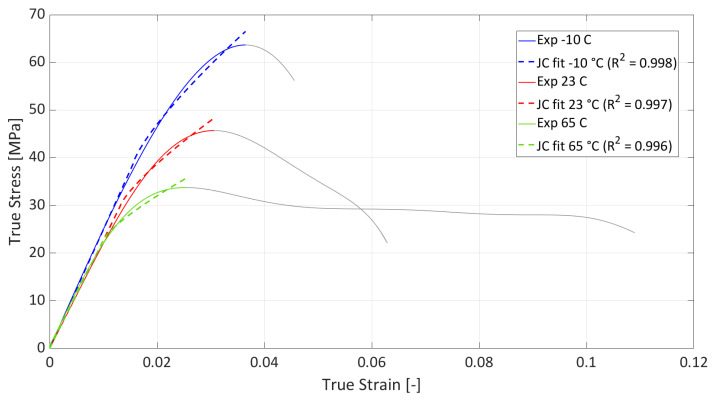
Globally fitted J-C parameters for each temperature, including *R*^2^.

The agreement between the curves of the global J-C model and the experimental curves (including *R*^2^) is shown in Figure 5. For a better understanding of the limitations of this model, the entire elastoplastic range is shown. The J-C model is a hardening model, so it only describes the area from the yield point to the ultimate strength and is not capable of modeling softening. The elastic part of the curve is described by Young’s modulus in the material model.

## 4. Finite Element Implementation

**Figure 6:**
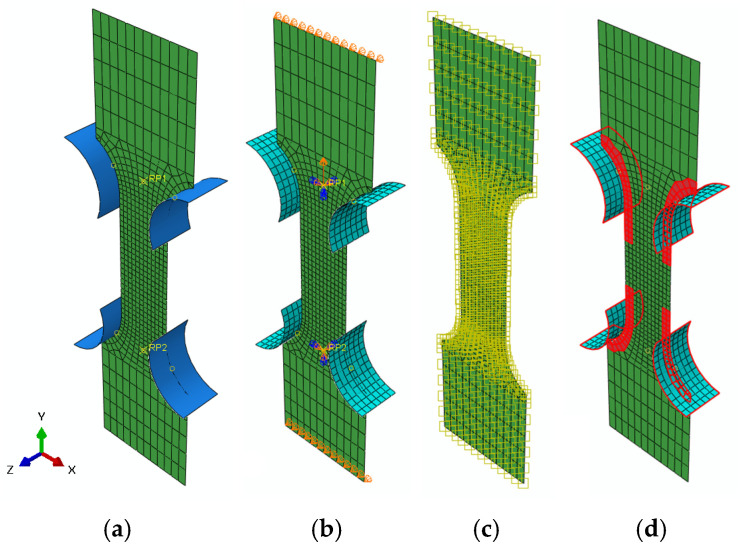
(**a**) FEM model; (**b**) boundary conditions; (**c**) temperature field; (**d**) contact.

Abaqus was used to numerically simulate the uniaxial tensile tests. The specimen was modeled using a two-dimensional shell element (Figure 6). The total number of elements in the sample model was 652, including 636 linear quadrilateral elements of type S4 and 16 linear triangular elements of type S3R. The approximate element size in the narrow part of the sample was 0.5 mm. The boundary conditions replicated the experimental setup. One end was fully constrained in RP2, and the opposite end was subjected to prescribed linear displacement (≈loading speed 0.17 mm/s) in RP1. Points RP1 and RP2 were connected to the upper and lower clamps, respectively. The clamps were modeled as discrete rigid bodies. A temperature field was predefined on the sample (only for simulations with global J-C parameters), and contact was ensured by means of general contact between the pins’ surface and the elements on the sample in the surrounding area of the pins. The contact interaction was defined using a penalty friction formulation with a friction coefficient of 0.3. Normal contact behavior allowed for separation between the contacting surfaces. In terms of simulation, this was a transient explicit dynamics problem.

## 5. Results and Discussion

**Figure 7:**
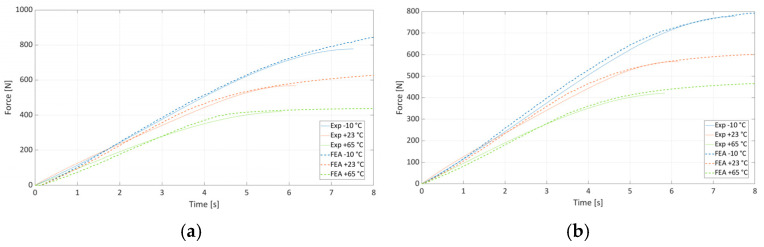
Comparison of experimental and numerical results: (**a**) individually fitted J-C parameters for each temperature; (**b**) globally fitted J-C parameters.

For all three temperatures, individually and globally, the J-C model exhibited very good agreement (Figure 7) with experimental force–time data. Residuals remained small across the entire hardening region.

The yield stress A decreased with increasing temperature, reflecting thermally activated softening. Parameter B, associated with hardening modulus, also varied systematically with temperature. The hardening rate parameter n showed temperature-dependence corresponding to molecular mobility changes in the polymer. These trends provide insight into the thermo-mechanical behavior of ASA. These observations align well with established polymer deformation theory.

Finite element simulations reproduced experimental tensile responses with a high correlation for all temperatures. Overlayed curves demonstrated good agreement up to the onset of necking. The deviations between the numerical and experimental curves in Figure 7 do not exceed 10%, and considering the dispersion in the tensile test diagrams in Figure 3, these differences can be considered fully acceptable. In terms of the accuracy of an individual J-C model when optimized to a specific temperature and the global J-C model, both models are capable of simulating the mechanical response of the material with high accuracy. The advantage of the global J-C model is that it can approximate the material response in the measured temperature range, so it is sufficient to define the temperature field for a given material in the numerical simulation. The individual J-C model can only be used for simulation at the constant temperature for which it was optimized.

**Figure 8:**
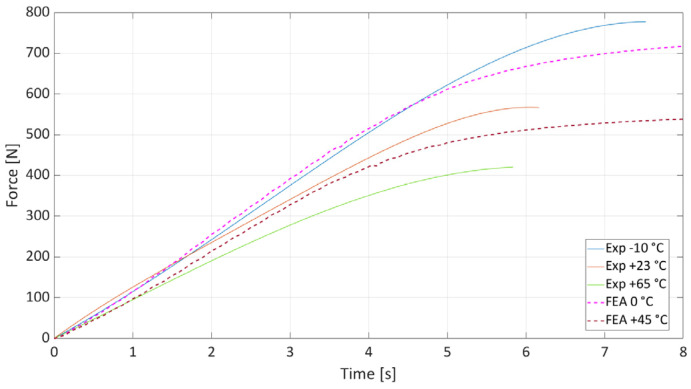
Validation of global J-C model at temperatures of 0 °C and +45 °C.

As part of the validation of the material model, the ability of the global J-C model to describe the behavior of the material between the tested temperatures was also examined. Temperatures of 0 °C and +45 °C were selected for this purpose. It was assumed that if the model worked correctly, the resulting dependencies would lie within the range bounded by the curves from the measurements. As can be seen in Figure 8, this assumption was confirmed. The results prove that the model is capable of approximating the behavior of the material in a wide range of temperatures below the glass transition temperature.

## 6. Conclusions

This study addressed the characterization and numerical modeling of the temperature-dependent plastic behavior of acrylonitrile styrene acrylate (ASA) within a temperature range relevant to engineering applications below the glass transition temperature. Uniaxial tensile tests performed at −10 °C, +23 °C, and +65 °C provided a comprehensive experimental dataset capturing the influence of temperature on stiffness, yield behavior, and plastic-hardening. Based on these data, Johnson–Cook plasticity parameters were identified using an inverse calibration procedure implemented in MATLAB.

Both individually calibrated and globally optimized Johnson–Cook models demonstrated very good agreement with experimental stress–strain responses. The finite element simulations conducted in Abaqus successfully reproduced the measured tensile behavior across all investigated temperatures, with deviations remaining within acceptable limits considering the natural dispersion of the polymer test data. The results confirm that, despite its original development for metals, the Johnson–Cook hardening formulation can effectively describe the monotonic plastic response of ASA under quasi-static loading conditions when appropriately calibrated.

It has been shown that the proposed global calibration strategy enables a continuous description of temperature-dependent plastic behavior using a single parameter set, offering a practical advantage for simulations involving spatially or temporally varying temperature fields. Validation at intermediate temperatures further confirmed the predictive capability of the global model within the investigated temperature range.

Overall, the presented methodology provides a robust and computationally efficient framework for the constitutive modeling of an ASA suitable for engineering-scale finite element analyses. The approach can be readily extended to other amorphous thermoplastics and forms a solid basis for future developments incorporating strain-rate effects, multiaxial loading conditions, and more advanced hardening or damage formulations. Additional constitutive formulations combining Voce and power-law hardening could further improve accuracy at large strains.

## Figures and Tables

**Table 1:** Young’s modulus of elasticity in tension.

Temperature [°C]	Young’s Modulus E [MPa]	Fit Quality *R*^2^ [-]
−10	2487 ± 196	0.999
+23	2206 ± 103	0.998
+65	2102 ± 156	0.996

**Table 2:** Young’s modulus used in FEA.

Temperature [K]	Young’s Modulus E [MPa]
263	1950
296	1650
338	1400

**Table 3:** Resulting individual J-C parameters for each temperature.

Temperature [K]	Parameter A [MPa]	Parameter B [MPa]	Parameter n [-]	Fit Quality *R*^2^ [-]
263	4.1	136.8	0.17	0.959
296	28.1	70.1	0.29	0.979
338	26.6	50.2	0.37	0.965

**Table 4:** Global J-C parameters for ASA.

Parameter A [MPa]	Parameter B [MPa]	Parameter n [-]	Parameter m [-]	Parameter T_melt_ [K]	Parameter T_0_ [K]
10	83.3	0.1	0.654	533	263

## Data Availability

The original contributions presented in this study are included in the article. Further inquiries can be directed to the corresponding author.

## References

[1] Siviour C.R., Jordan J.L. (2016). High strain rate mechanics of polymers: A review. J. Dyn. Behav. Mater..

[2] Field J.E., Walley S.M., Proud W.G., Goldrein H.T., Siviour C.R. (2004). Review of experimental techniques for high rate deformation and shock studies. Int. J. Impact Eng..

[3] Crawford R.J., Martin P.J. (2020). Plastics Engineering.

[4] Bonten C. (2019). Plastics Technology, Introduction and Fundamentals.

[5] Zhu S., Yang Y., Pan W., Liu J., Li Y., Wei Z., Sang L., Leng X. (2026). Preparation of ASA resin with outstanding mechanical properties and processing liquidity and its application in malleable devices. Eur. Polym. J..

[6] Huang W., Mao Z., Xu Z., Xiang B., Zhang J. (2019). Synthesis and characterization of size-tunable core-shell structural polyacrylate-graft-poly(acrylonitrile-ran-styrene) (ASA) by pre-emulsion semi-continuous polymerization. Eur. Polym. J..

[7] Zatloukal J., Viry M., Mizera A., Stoklásek P., Miškařík L., Bednařík M. (2025). Optimizing Interfacial Adhesion and Mechanical Performance of Multimaterial Joints Fabricated by Material Extrusion. Materials.

[8] Appalsamy T., Hamilton S.L., Kgaphola M.J. (2024). Tensile Test Analysis of 3D Printed Specimens with Varying Print Orientation and Infill Density. J. Compos. Sci..

[9] Zhao W., Steinmann P., Pfaller S. (2024). Modeling steady state rate- and temperature-dependent strain hardening behavior of glassy polymers. Mech. Mater..

[10] Richeton J., Ahzi S., Vecchio K.S., Jiang F.C., Adharapurapu R.R. (2006). Influence of temperature and strain rate on the mechanical behavior of three amorphous polymers: Characterization and modeling of the compressive yield stress. Int. J. Solids Struct..

[11] Barba D., Arias A., Garcia-Gonzalez D. (2020). Temperature and strain rate dependences on hardening and softening behaviors in semi-crystalline polymers: Application to PEEK. Int. J. Solids Struct..

[12] Richeton J., Ahzi S., Vecchio K.S., Jiang F.C., Makradi A. (2007). Modeling and validation of the large deformation inelastic response of amorphous polymers over a wide range of temperatures and strain rates. Int. J. Solids Struct..

[13] Zrida M., Laurent H., Grolleau V., Rio G., Khlif M., Guines D., Masmoudi N., Bradai C. (2010). High-speed tensile tests on a polypropylene material. Polym. Test..

[14] Huang P.Y., Guo Z.S., Feng J.M. (2020). General model of temperature-dependent modulus and yield strength of thermoplastic polymers. Chin. J. Polym. Sci..

[15] Yang M., Li W., Dong P., Ma Y., He Y., Zhao Z., Chen L. (2022). Temperature and strain rate sensitivity of yield strength of amorphous polymers: Characterization and modeling. Polymer.

[16] Voce E. (1948). The relationship between stress and strain for homogeneous deformation. J. Inst. Met..

[17] Voce E. (1955). A practical strain hardening function. Metallurgia.

[18] Johnson G.R., Cook W.H. (1985). Fracture Characteristics of Three Metals Subjected to Various Strains, Strain rates, Temperatures and Pressures. Eng. Fract. Mech..

[19] Jang T.J., Kim J.B., Shin H. (2021). Identification of plastic constitutive Johnson–Cook model parameters by optimization-based inverse method. J. Comput. Des. Eng..

[20] Dura H.B., Hazell P.J., Wang H., Escobedo-Diaz J.P., Wang J. (2025). Strain rate sensitivity of five 3D printed polymer materials. Polymer.

[21] Zelepugin S.A., Cherepanov R.O., Pakhnutova N.V. (2023). Optimization of Johnson–Cook Constitutive Model Parameters Using the Nesterov Gradient-Descent Method. Materials.

[22] O’Toole L., Haridas R.S., Mishra R.S., Fang F. (2023). Determination of Johnson-Cook plasticity model parameters for CoCrMo alloy. Mater. Today Commun..

[23] Zhou J., Xia Z., Ma D., Wang H. (2024). Study of Dynamic Failure Behavior of a Type of PC/ABS Composite. Materials.

[24] Chen F., Ou H., Lu B., Long H. (2016). A constitutive model of polyether-ether-ketone (PEEK). J. Mech. Behav. Biomed. Mater..

[25] Pradeep S.A., Iyer R.K., Kazan H., Pilla S. (2017). Applied Plastics Engineering Handbook.

[26] Yap Y.L., Toh W., Koneru R., Chua Z.Y., Lin K., Yeoh K.M., Lim C.M., Lee J.S., Plemping N.A., Lin R. (2019). Finite element analysis of 3D-Printed Acrylonitrile Styrene Acrylate (ASA) with Ultrasonic material characterization. Int. J. Comput. Mater. Sci. Eng..

[27] Guessasma S., Belhabib S., Nouri H. (2019). Microstructure, Thermal and Mechanical Behavior of 3D Printed Acrylonitrile Styrene Acrylate. Macromol. Mater. Eng..

[28] Karkalos N.E., Rydzoń K., Papazoglou E.L., Karmiris-Obratański P. (2024). Analyzing the effect of infill density on the mechanical compression of ASA in additive manufacturing: A FEM perspective. Int. J. Adv. Manuf. Technol..

[29] Garzon-Hernandez S., Arias A., Garcia-Gonzalez D. (2020). A continuum constitutive model for FDM 3D printed thermoplastics. Compos. Part B Eng..

[30] Jiquan L., Yadong J., Taidong L., Zhou Z., Hangchao Z., Xiang P., Shaofei J. (2020). Tensile Behavior of Acrylonitrile Butadiene Styrene at Different Temperatures. Adv. Polym. Technol..

[31] Wang H., Zhang Y., Huang Z., Tang Z., Wang Y., Zhou H. (2018). Establishment and comparison of four constitutive relationships of PC/ABS from low to high uniaxial strain rates. Mech. Time-Depend. Mater..

[32] BASF Aktiengesellschaft (2003). Luran^®^ S 778 T (ASA): Product Information.

[33] Dassault Systèmes (2024). Abaqus Documentation, Version 2024.

[34] Biron M. (2016). 8-Thermal Properties. Material Selection for Thermoplastic Parts.

